# Optimized Flux Single-Crystal Growth of the Quantum Spin Liquid Candidate NdTa_7_O_19_ and Other Rare-Earth Heptatantalates, ErTa_7_O_19_ and GdTa_7_O_19_

**DOI:** 10.1021/acs.cgd.5c00624

**Published:** 2025-06-02

**Authors:** Lia Šibav, Matic Lozinšek, Zvonko Jagličić, Tina Arh, Panchanana Khuntia, Andrej Zorko, Mirela Dragomir

**Affiliations:** † 61790 Jožef Stefan Institute, Jamova cesta 39, 1000 Ljubljana, Slovenia; ‡ Jožef Stefan International Postgraduate School, Jamova cesta 39, 1000 Ljubljana, Slovenia; § Faculty of Civil and Geodetic Engineering, University of Ljubljana, Jamova cesta 2, 1000 Ljubljana, Slovenia; ∥ Institute of Mathematics, Physics and Mechanics, Jadranska cesta 19, 1000 Ljubljana, Slovenia; ⊥ Faculty of Mathematics and Physics, University of Ljubljana, 1000 Ljubljana, Slovenia; # Department of Physics, Indian Institute of Technology Madras, 600036 Chennai, India; ¶ Quantum Centre of Excellence for Diamond and Emergent Materials, Indian Institute of Technology Madras, 600036 Chennai, India

## Abstract

Single crystals are essential for characterizing a wide range of magnetic states, including exotic ones such as quantum spin liquids. This study reports a flux method for growing single crystals of NdTa_7_O_19_, the first quantum spin liquid candidate on a triangular spin-lattice with dominant Ising-like spin correlations. Purple NdTa_7_O_19_ single crystals with hexagonal morphology were successfully grown by using a K_2_Mo_3_O_10_–B_2_O_3_ flux. With lateral sizes up to 3.5 mm and a thickness up to 2 mm, these are the largest dimensions reported to date. The chemical composition was confirmed by powder and single-crystal X-ray diffraction along with scanning electron microscopy with energy dispersive X-ray spectroscopy. Aiming for an accurate determination of the magnetic anisotropy and its effect on the magnetic properties, NdTa_7_O_19_ crystals were additionally analyzed by magnetic susceptibility, revealing a substantial anisotropy without long-range magnetic ordering down to 2 K. Single crystals of two novel rare-earth heptatantalates, ErTa_7_O_19_ and GdTa_7_O_19_, were also grown, and their magnetic properties investigated. The magnetic anisotropy of ErTa_7_O_19_ closely resembles that of isostructural NdTa_7_O_19_, indicating the possibility of a similar exotic magnetic ground state. In contrast, GdTa_7_O_19_ shows paramagnetic behavior, consistent with previous results obtained for polycrystalline samples.

## Graphical abstract

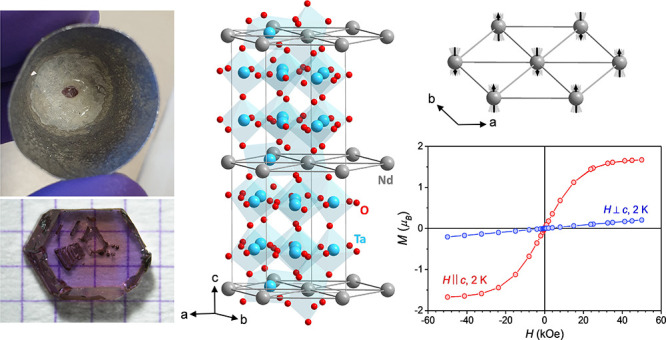

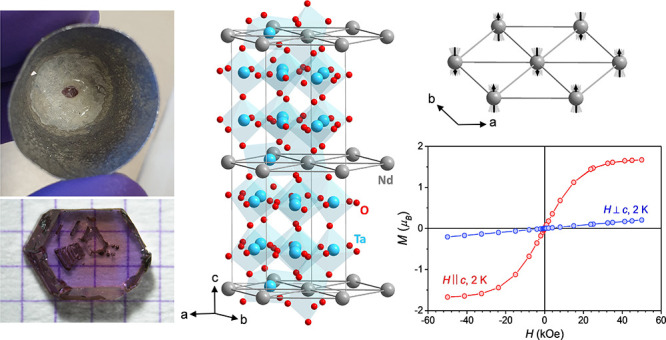

## 1 Introduction

Rare-earth heptatantalates, abbreviated as RETa_7_O_19_, with RE representing a trivalent rare-earth element, are promising materials for nonlinear optics and laser technology due to their noncentrosymmetric structure as well as high chemical and thermal stability, high concentration of optically active ions, high mechanical strength, and high thermal conductivity.^1–4^ Among this series, NdTa_7_O_19_ is regarded as the most auspicious candidate for such applications.^2,4^

Recently, NdTa_7_O_19_ has also been identified as a very intriguing magnetic material, more specifically, a potential triangular antiferromagnet with an Ising quantum spin liquid ground state.^5^ Low-dimensional antiferromagnets realized on a perfect triangular lattice, such as NdTa_7_O_19_, are highly desired as a large magnetic anisotropy could explain a possible quantum spin liquid ground state.^6,7^ Indication of a spin liquid ground state in NdTa_7_O_19_ was found via absence of magnetic Bragg peaks in neutron powder diffraction at temperatures down to 40 mK,^5^ the presence of magnetic diffuse scattering, and persistent, low-temperature spin dynamics detected via muon spectroscopy, thus combining most suitable experimental techniques that can be used to detect such elusive states.^8^ The splitting of crystal-electric field levels and bulk magnetic measurements on this material further suggested the presence of large magnetic anisotropy.^5^ However, since these measurements were conducted on polycrystalline NdTa_7_O_19_, a more precise determination of magnetic anisotropy is dependent on the availability of single crystals.^9–11^ Single crystals would also allow for more refined magnetic measurements, e.g., enabling wave-vector-resolved insight into spin correlations or the detection of the anticipated fractionalized excitations. Furthermore, given that materials with frustrated lattices and enhanced quantum properties tend to be particularly sensitive to defects (e.g., chemical disorder, undesirable impurities, etc.),^8,11^ there is a clear preference for high-quality single crystals of NdTa_7_O_19_ over polycrystalline samples, where such defects are minimized.

Single crystals of NdTa_7_O_19_ have been previously grown for optical applications by a flux method using two different fluxes, Li_2_B_4_O_7_ ^1,3^ and K_2_Mo_3_O_10_—with a small addition of B_2_O_3_.^3,4^ The literature reports showed a preference for starting from the constituent oxides Nd_2_O_3_ and Ta_2_O_5_ ^1–4^ instead of polycrystalline NdTa_7_O_19_, which could be due to the fact that solid-state synthesis resulted in nonstoichiometric powders.^4^ The largest crystals grown from the oxides were limited to 1.5 mm in lateral size.^12^ Starting with constituent oxides is, however, less advantageous than starting from a polycrystalline powder of the correct composition. Different dissolution rates of the constituent oxides affect concentrations of reagents in the melts and drive the melt into a different supersaturated regime, from which nucleation of undesired phases takes place.^13^ Indeed, the phase diagrams showed a narrow primary crystallization region of the NdTa_7_O_19_ phase as well as multiple regions of formation of secondary phases, which included Nd_0.33_TaO_3_ and NdTaO_4_.^1,3,4^ This could also be the reason why these reports^1–4^ only provide rough guidelines rather than exact protocols required for the growth of high-quality, millimeter-sized single crystals.

With the aim of growing NdTa_7_O_19_ crystals of suitable size for magnetic measurements and other techniques that require larger crystals, such as neutron scattering or muon spectroscopy, and of establishing a more precise protocol that could be extended to other rare-earth members of the RETa_7_O_19_ family, in this study, we chose in this study to explore a flux growth method using K_2_Mo_3_O_10_–B_2_O_3_ as the flux.^14^ This flux has a high dissolution ability due to the high chemical activity of alkali polymolybdates, as well as a relatively low melting temperature.^4^ The presence of small amounts of B_2_O_3_ increases the solubility, decreases the saturation temperature, and reduces the flux volatility without significantly altering the flux viscosity.^15^ These benefits have previously been successfully exploited for single-crystal growth of other systems such as rare-earth borates REAl_3_(BO_3_)_4_ ^16–22^ or rare-earth phosphates.^23^

In this study, nearly single-phase polycrystalline NdTa_7_O_19_ was successfully prepared. A previously reported solid-state method^24^ was optimized, resulting in a 98(1) wt % of the main phase, which was used as a starting material for flux growth of NdTa_7_O_19_ single crystals of predominantly hexagonal, plate-like morphologies with lateral sizes up to 3.5 mm and thicknesses up to 2 mm—the largest NdTa_7_O_19_ single crystals reported to date. The flux method presented here was further employed to grow single crystals of two other novel rare-earth heptatantalates, ErTa_7_O_19_ and GdTa_7_O_19_. The composition and morphology of all newly grown crystals were characterized by powder X-ray diffraction (PXRD) and single-crystal X-ray diffraction (SCXRD) as well as scanning electron microscopy (SEM) coupled with energy dispersive X-ray spectroscopy (EDS). Furthermore, magnetic susceptibility measurements were employed to investigate the magnetic anisotropy.

## 2 Experimental Section

### 2.1 Solid-State Synthesis

Three polycrystalline RETa_7_O_19_ members, NdTa_7_O_19_, ErTa_7_O_19_, and GdTa_7_O_19_, were synthesized by a solid-state reaction similar to a previously reported procedure.^24^ Stoichiometric amounts of Nd_2_O_3_ (Thermo Scientific, 99.99%), Er_2_O_3_ (Thermo Scientific, 99.99%), and Gd_2_O_3_ (Thermo Scientific, 99.99%), together with Ta_2_O_5_ (Alpha Aesar, 99.85 or 99.99%), were weighed, hand-homogenized in an agate mortar, and pressed into pellets with a 10 mm diameter using a force of 50 kN. Prior to the synthesis, all three rare-earth oxides were preannealed at 1000 °C for 24 h. Multiple synthesis cycles (5–9) were performed at temperatures *T* = 900–1200 °C with intermediate regrinding, resulting in about 8.5 g (5 mmol) of the polycrystalline product. As the main flux component, potassium molybdate (K_2_Mo_3_O_10_, about 30 mmol), is not commercially available, it was synthesized using K_2_CO_3_ (Chempur, 99.9%) and MoO_3_ (Merck, 99.9%) by a solid-state reaction at 500 °C, for two cycles of 72 h each, with intermediate grinding (eq 1)­

1 $$K_{2}CO_{3}+3MoO_{3}\to K_{2}Mo_{3}O_{10}+CO_{2}↑$$

### 2.2 Single-Crystal Growth

Polycrystalline RETa_7_O_19_ samples, obtained by solid-state reactions, were used as starting materials for flux-growth experiments, employing a K_2_Mo_3_O_10_–B_2_O_3_ flux. In each growth experiment, polycrystalline RETa_7_O_19_, K_2_Mo_3_O_10_, and B_2_O_3_ were weighed in the predetermined mass ratio, hand-homogenized in an agate mortar, and placed into platinum (Pt) crucibles, which were placed into alumina crucibles covered by alumina caps (Figure 1). For the NdTa_7_O_19_ single-crystal growth experiments, Pt crucibles with a volume of 5 mL were used, while ErTa_7_O_19_ and GdTa_7_O_19_ growths were performed in 10 mL Pt crucibles.

**Figure 1:**
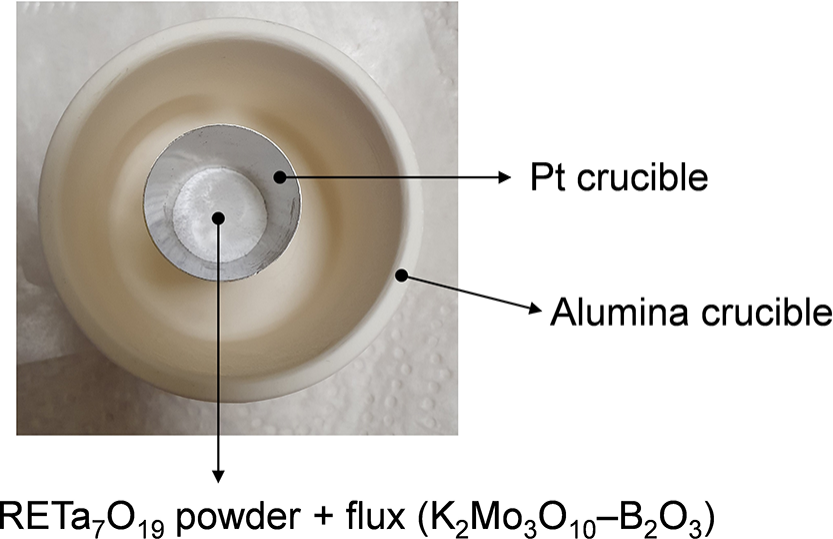
Experimental setup for single-crystal growth, showing a platinum crucible containing the reaction mixture of NdTa_7_O_19_ and the K_2_Mo_3_O_10_–B_2_O_3_ flux, placed inside an alumina crucible, which was covered with an alumina cap.

In NdTa_7_O_19_ single-crystal growth, cooling was done in two steps; first, a slower cooling step of 0.5, 1, or 2 °C/h was employed, followed by a rapid cooling step of 10 or 50 °C/h (Table 1) with a minimal dwell time at the transition temperature in between the steps. At the end of rapid cooling, the reaction mixture was allowed to cool naturally. The crystals were separated from the crucible by using deionized water in an ultrasonic bath.

**Table 1:** Summary of Tested Parameters for NdTa_7_O_19_ Flux Growth

parameter	tested conditions
batch size (g)	0.6, 0.7, 0.9, 1, 1.1, 1.2, 1.6, 2.2, 3, 4.4
flux-to-material mass ratio	5:1, 8:1, 9:1, 10:1, 15:1
flux composition (K_2_Mo_3_O_10_–B_2_O_3_ mass ratio)	2.3:1, 4:1, 8:1, 9:1, 99:1
dwell temperature (°C)	700, 1100, 1150, 1200
dwell time (h)	2, 24, 48
slower cooling rate (°C/h)	0.5, 1, 2
transition temperature (°C)	500, 950
rapid cooling rate (°C/h)	10, 50

The following growth parameters were optimized for NdTa_7_O_19_: (i) batch size, (ii) flux-to-material mass ratio, (iii) flux composition (K_2_Mo_3_O_10_–B_2_O_3_ mass ratio), (iv) dwell temperature, (v) dwell time, and (vi) cooling rate. These parameters were varied and optimized in order to grow the largest number of NdTa_7_O_19_ single crystals (Table 1). The optimal conditions were slightly modified for subsequent growth experiments of ErTa_7_O_19_ (Table 2). The dwell time, dwell temperature, transition temperature, and rapid cooling rate were kept constant at 24 h, 1100 °C, 950 and 10 °C/h.

**Table 2:** Summary of Tested Parameters for ErTa_7_O_19_ Flux Growth

parameter	tested conditions
batch size (g)	0.8, 0.9, 1.1, 1.22, 1.35, 2.2
flux-to-material mass ratio	6:1, 7:1, 8:1, 9:1, 10:1
flux composition (K_2_Mo_3_O_10_–B_2_O_3_ mass ratio)	3:1, 4:1, 5:1, 9:1
slow cooling rate (°C/h)	0.3, 0.5, 0.7, 0.8. 1, 2, 5

Flux growth of GdTa_7_O_19_ was performed using a 4:1 K_2_Mo_3_O_10_–B_2_O_3_ mass ratio, an 8:1 flux-to-material mass ratio, and a batch size of 0.90 g. Two slow cooling rates were tested, 0.8 and 0.5 °C/h. The remaining parameters of the temperature profile were identical to those employed for ErTa_7_O_19_ growth.

### 2.3 Powder X-ray Diffraction

Polycrystalline RETa_7_O_19_ samples obtained by solid-state synthesis were first examined by PXRD using a Rigaku MiniFlex600-C Benchtop XRD with Cu Kα radiation in the 2θ range 3–120° with a step size of 0.010° and a speed of 2.5°/min. The primary flux component, K_2_Mo_3_O_10_, was also examined using the same conditions, albeit with a smaller 2θ range of 3–90°. Phase identification was performed using SmartLab Studio II software. The Rietveld refinements were performed using the least-squares method in GSAS-II.^25^

### 2.4 Single-Crystal X-ray Diffraction

The grown crystals were first examined under a polarizing microscope. Selected crystals of appropriate sizes were mounted on MiTeGen Dual Thickness MicroLoops with Baysilone-Paste (Bayer-Silicone, Mittelviskos) and measured on a Rigaku OD XtaLAB Synergy-S Dualflex diffractometer equipped with a PhotonJet-S microfocus Ag Kα X-ray source and an Eiger2 R CdTe 1M hybrid-photon-counting detector. *CrysAlisPro* software^26^ was employed for data collection and reduction. Crystal structures were solved by *olex*2.*solve* and refined by *SHELXL* ^27^ within *OLEX*2 program.^28^

### 2.5 Laue Diffraction

White beam X-ray backscatter diffraction was used to assess the surface quality and the crystallographic orientation of the single crystals. A real-time Laue system (Laue-Camera GmbH) was used with an X-ray Seifert ID3003 generator equipped with an MWL 120 detector.

### 2.6 SEM and EDS

For the SEM analyses, a few small NdTa_7_O_19_ single crystals were placed on carbon tape and carbon coated using a Balzers SCD 050 sputter coater. The imaging and compositional analyses were performed on a Thermo Fisher Quanta 650 ESEM instrument equipped with an energy-dispersive X-ray spectrometer–EDS (Oxford Instruments, AZtec Live, Ultim Max SDD 65 mm^2^). The accelerating voltage used was 20 kV in all cases.

### 2.7 Magnetic Susceptibility

The magnetic susceptibility measurements were carried out using an MPMS-XL-5 SQUID magnetometer from Quantum Design in the 2–300 K temperature range in an applied magnetic field of 1 kOe. A single crystal of NdTa_7_O_19_ (16 mg), ErTa_7_O_19_ (4 mg), and GdTa_7_O_19_ (2 mg) was glued to a straw with diamagnetic Apiezon N grease in different orientations to the external magnetic field. The field dependence of the isothermal magnetization was measured between −50 and 50 kOe at 2 K.

## 3 Results and Discussion

### 3.1 Solid-State Synthesis

The primary flux component, K_2_Mo_3_O_10_, was synthesized using a solid-state thermal method according to eq 1, as it is not commercially available. Rietveld refinement analysis on the resulting polycrystalline K_2_Mo_3_O_10_ showed 82.0(4) wt % of the main phase (*C*2/*c* space group), 9.2(2) wt % of K_2_Mo_4_O_13_, and 8.8(3) wt % of K_2_Mo_2_O_7_ (Figure 2a). The additional molybdates were likely formed by the loss of potassium. This was subsequently optimized to an almost pure phase, 98(1) wt %, K_2_Mo_3_O_10_. However, the crystal growths were performed using the batch presented in Figure 2a.

**Figure 2:**
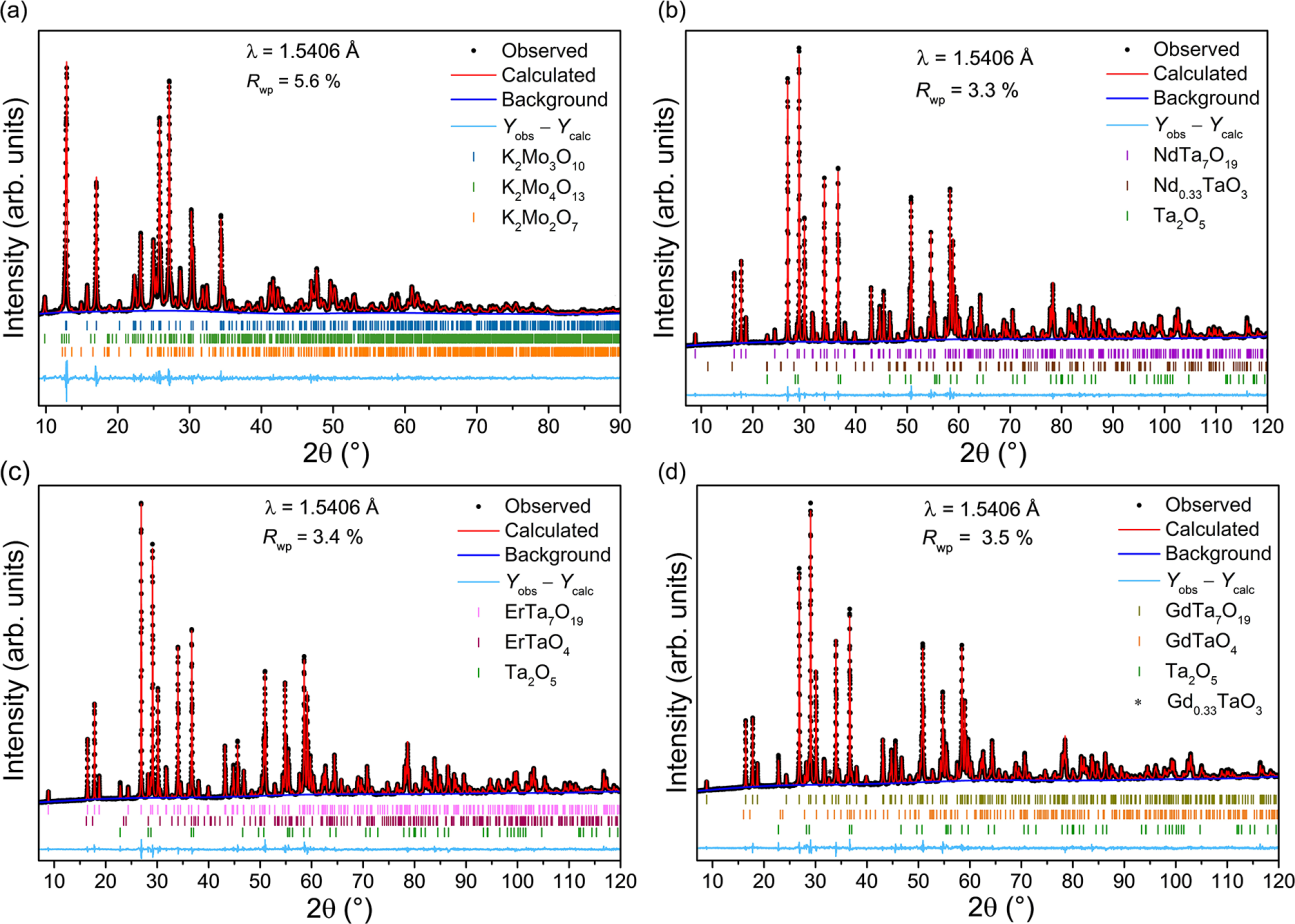
Rietveld refinement profiles of laboratory PXRD data (Cu Kα, *T* = 293 K) of (a) the primary flux component, K_2_Mo_3_O_10_, (b) NdTa_7_O_19_, (c) ErTa_7_O_19_, and (d) GdTa_7_O_19_. The Bragg 2θ positions are marked as bars below each diffractogram.

As mentioned in the previous section, this study aimed to perform single-crystal growth experiments starting from polycrystalline RETa_7_O_19_ to avoid the secondary phases previously reported in the literature.^1–4^ Thus, the three phases NdTa_7_O_19_, ErTa_7_O_19_, and GdTa_7_O_19_ were first prepared by solid-state synthesis. The phase identifications and Rietveld refinement analyses of the powder data indicated that the three members of the series are isostructural and adopt the *P*6̅*c*2 space group, in good agreement with the literature (Figure 2b–d).^29,30^ Detailed results of the Rietveld refinement analysis are available in the Supporting Information, Table S1. A 0.6% decrease in the unit cell volume is observed from Nd (*V* = 668.536(4) Å^3^) to Gd (*V* = 664.780(7) Å^3^) and a 1% reduction to the Er member (*V* = 661.418(5) Å^3^), as expected following the decreasing radii of the rare-earth ions due to the lanthanoid contraction.^31^ A parallel trend is observed with the magnetic intralayer RE–RE distance, which decreases from 6.2228(1) Å for Nd to 6.2119(1) Å for Gd and 6.2018(1) Å for the Er member, while the magnetic interlayer distances decrease from 9.9678(1) Å for Nd to 9.9465(1) Å for Gd and 9.9285(1) Å for the Er member.

A small fraction of secondary phases was observed in the powder X-ray diffractograms of each heptatantalate (Figure 2b–d), identified as unreacted Ta_2_O_5_ and two additional rare-earth tantalates. The Rietveld refinement analysis revealed 1.2(2) wt % of Ta_2_O_5_ and 1.2(1) wt % of Nd_0.33_TaO_3_ for the NdTa_7_O_19_ sample, while the ErTa_7_O_19_ sample contained 5.1(1) wt % of Ta_2_O_5_ and 2.2(1) wt % of ErTaO_4_, with the GdTa_7_O_19_ sample containing a slighter larger content of impurities, namely, 7.9(1) wt % Ta_2_O_5_, 1.6(1) wt % GdTaO_4_, and 1.0(1) wt % Gd_0.33_TaO_3_. One way to minimize the amount of these phases is to increase the annealing temperature in the solid-state reactions and the dwell time. Despite the small amount of impurities, the powders were used for single-crystal growth, as the process can also serve as a purification method, ensuring no significant impact on the study.

### 3.2 Single-Crystal Growth

The optimized single-crystal growth procedure employed in this study resulted in purple transparent NdTa_7_O_19_ crystals of predominantly hexagonal morphologies (Figure 3a), which measured up to 3.5 mm in lateral size and up to 2 mm in thickness.

**Figure 3:**
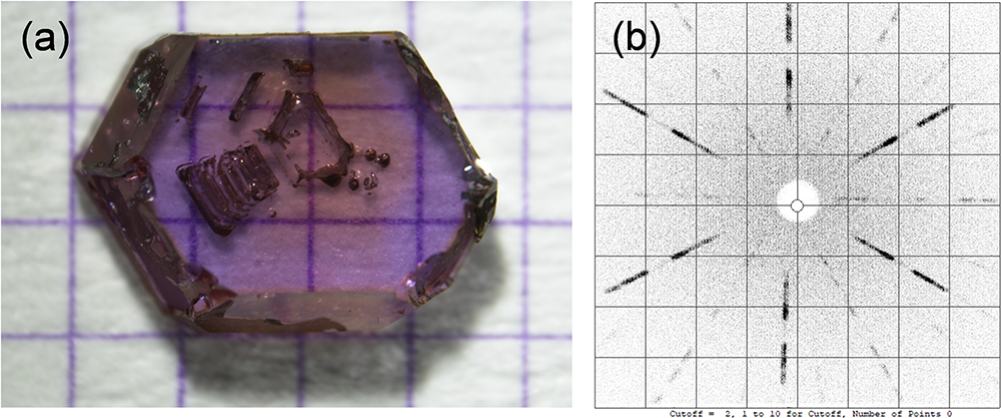
(a) Hexagonal NdTa_7_O_19_ crystal grown in this study on a millimeter grid and (b) corresponding X-ray Laue back-reflection pattern observed along the *c* crystallographic direction, displaying the 6-fold symmetry.

The orientation of the crystals^1–4,12^ —with hexagonal planes being parallel to the *ab* plane and perpendicular to the *c*-crystallographic direction (Figure 3a)—was confirmed by Laue X-ray diffraction (Figure 3b).

The optimization of the temperature profile for NdTa_7_O_19_ flux growth identified the dwell temperature and the slow cooling rate as pivotal parameters (Figure 4). The optimal dwell temperature was determined to be 1100 °C. While previous studies^2,4^ reported higher dwell temperatures of 1150 and 1200 °C, in the present study, these elevated temperatures resulted in increased flux volatility, which led to the formation of numerous nucleation sites and, consequently, smaller single crystals (see Supporting Information, Figure S2 for pictures of the resulting crystals). The optimized cooling rate of the first step, which yielded the largest crystal sizes, was 0.5 °C/h. Higher cooling rates of 1 or 2 °C/h resulted in multiple smaller crystals. The dwell time was found to be a less significant parameter; observations indicated that a 48 h dwell time did not result in substantially different crystal sizes compared to a shorter 24 h dwell time.

**Figure 4:**
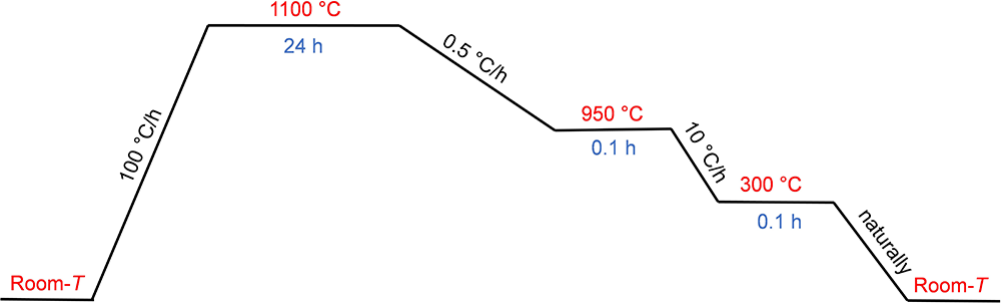
Optimal temperature profile for flux growth of NdTa_7_O_19_ using the K_2_Mo_3_O_10_–B_2_O_3_ flux. The dwell temperature, 1100 °C, and the cooling rate of the first step, 0.5 °C/h, were identified as critical crystal-growth parameters.

Other parameters tested during the growth process (displayed in Table 1) included the following: (i) the batch size, (ii) the flux-to-material mass ratio, and (iii) the flux composition.

(i) The optimal batch size was determined to be approximately 1 g. Attempts to increase crystal sizes by doubling or further increasing the mass proved ineffective, resulting only in the formation of multiple smaller crystals.
(ii) The optimal range of flux-to-material mass ratios was found to be between 8:1 and 10:1. Other ratios, including 5:1 and 15:1, were also investigated; however, they did not yield any large single crystals.
(iii) The flux composition also turned out to be a crucial parameter, being the primary factor affecting the crystal morphology, while additionally influencing the crystal size. Experiments with a K_2_Mo_3_O_10_–B_2_O_3_ mass ratio of 9:1 resulted in plate-like crystals. An increase of the B_2_O_3_ content in the K_2_Mo_3_O_10_–B_2_O_3_ mass ratio led to increased thicknesses of the crystals, transitioning from plate-like toward isometric morphologies (Supporting Information, Figure S1), which was also observed in previous literature reports.^2–4^

The optimal K_2_Mo_3_O_10_–B_2_O_3_ mass ratio of 4:1 yielded predominantly hexagonal crystals of approximately 1 mm thickness. Elongated morphologies, as previously reported in the literature,^2–4^ were not observed. Further experiments with higher or lower ratios (Table 1) failed to produce large single crystals (Figure S2). The optimal parameters for NdTa_7_O_19_ growth are summarized in Table 3.

**Table 3:** Optimal Growth Parameters for NdTa_7_O_19_

parameter	optimal conditions
batch size (g)	0.9–1.1
flux-to-material mass ratio	8:1–10:1
flux composition (K_2_Mo_3_O_10_–B_2_O_3_ mass ratio)	4:1
dwell temperature (°C)	1100
slow cooling rate (°C/h)	0.5

Seeding, which plays a crucial role in crystal growth, was also investigated in this study. A small crystal ('seed') obtained from a previous experiment was placed at the bottom of the crucible to hinder multiple nucleation.^32^ This method promotes controlled nucleation and facilitates the growth of high-quality single crystals with fewer defects. However, in our case, seeding had minimal impact on crystal size, indicating that it is not a critical factor in these growth processes.

The optimal NdTa_7_O_19_ crystal growth conditions were also employed for the growth of ErTa_7_O_19_ single crystals, with some optimization of the cooling rates (Table 2). The largest crystals were grown using a cooling rate of 0.8 °C/h, while lower rates of 0.5 and 0.3 °C/h resulted in increased flux volatility, as deduced from the mass loss.

The flux-to-material mass ratios that yielded the largest crystal sizes were 7:1 and 8:1, while the optimal batch size was found to be 0.8–0.9 g. Higher masses resulted in the formation of multiple smaller crystals. The optimal K_2_Mo_3_O_10_–B_2_O_3_ mass ratios ranged from 3:1 to 5:1. Similarly as in the NdTa_7_O_19_ growth, the crystal morphology was influenced by the flux composition. Specifically, a higher content of B_2_O_3_ in the K_2_Mo_3_O_10_–B_2_O_3_ flux resulted in decreased lateral sizes of the crystals and increased thicknesses. The optimal growth parameters are summarized in Table 4.

**Table 4:** Optimal Growth Parameters for ErTa_7_O_19_

parameter	optimal conditions
batch size (g)	0.8–0.9
flux-to-material mass ratio	7:1–8:1
flux composition (K_2_Mo_3_O_10_–B_2_O_3_ mass ratio)	3:1–5:1
dwell temperature (°C)	1100
slow cooling rate (°C/h)	0.8

Pink ErTa_7_O_19_ crystals exhibited predominantly hexagonal morphologies (Figure 5a) with lateral sizes of up to 2.5 mm and thicknesses of up to 0.5 mm. The orientation of ErTa_7_O_19_ crystals (Figure 5b) was confirmed by X-ray Laue diffraction (Figure 5c).

**Figure 5:**
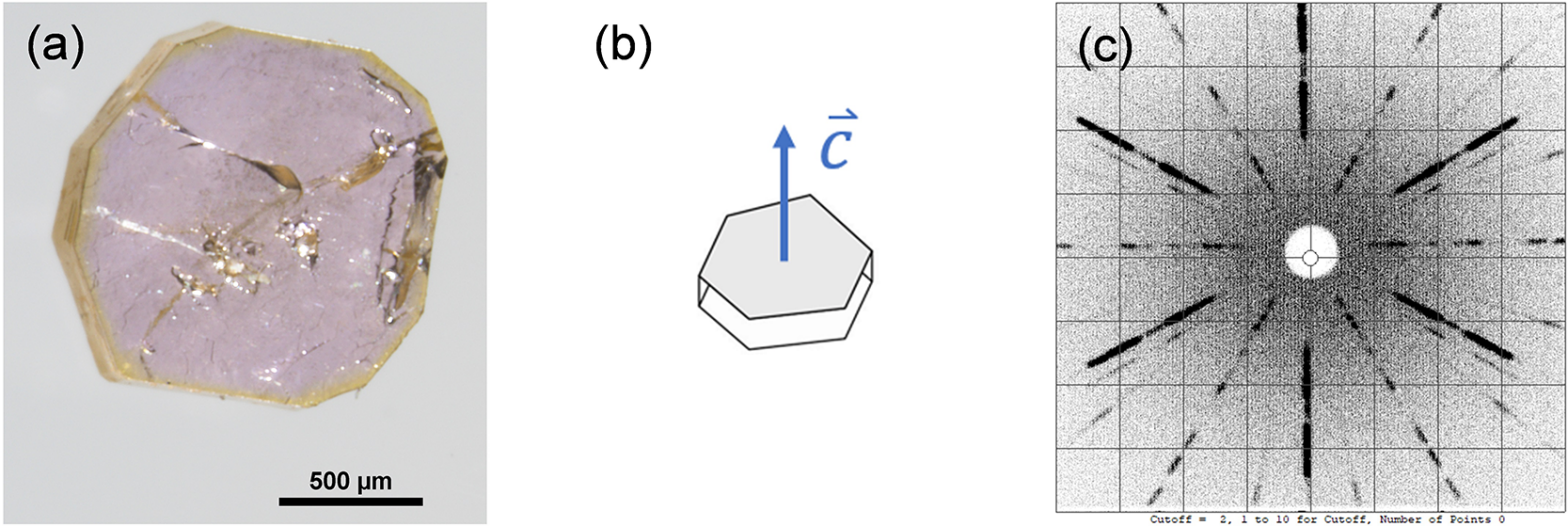
(a) ErTa_7_O_19_ single crystal grown by the flux method. (b) Expected orientation of the crystallographic *c*-axis of an ErTa_7_O_19_ crystal. (c) Corresponding X-ray Laue back-reflection pattern collected on an ErTa_7_O_19_ single crystal showing the 6-fold axis along the *c*-crystallographic direction.

For GdTa_7_O_19_ single-crystal growth, the optimal NdTa_7_O_19_ growth conditions were employed, as listed in Table 3. Similar to the other two representatives of the series, yellow GdTa_7_O_19_ single crystals exhibited pseudohexagonal morphologies with lateral sizes up to 4 mm and thicknesses up to 0.5 mm (Figure 6a–c).

**Figure 6:**
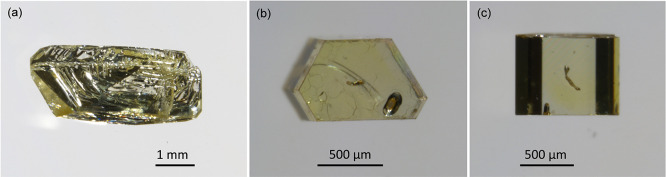
(a) Largest as-grown pseudohexagonal GdTa_7_O_19_ crystal. (b) Frontal view and (c) side view of another, smaller, hexagonal GdTa_7_O_19_ single crystal grown in this study.

### 3.3 Chemical Analysis

The grown single crystals of NdTa_7_O_19_, ErTa_7_O_19_, and GdTa_7_O_19_ were analyzed by PXRD and SCXRD. All structural information can be found in the Supporting Information, Tables S1–S6. All the structures were solved and refined in the *P*6̅*c*2 space group.^29^ Initially, the *P*6_3_/*mcm* space group was identified for CeTa_7_O_19_ and the RETa_7_O_19_ series.^33^ However, a year later, the same author reported a preference for the *P*6̅*c*2 space group.^34^

The RE ions form a perfect triangular lattice (side view: Figure 7a, top view: Figure 7b), where each RE ion is coordinated by eight oxygen ions, forming distorted REO_8_ polyhedra that share edges with neighboring TaO_6_ octahedra. The magnetic layers are separated by two nonmagnetic Ta layers composed of TaO_7_ polyhedra.

**Figure 7:**
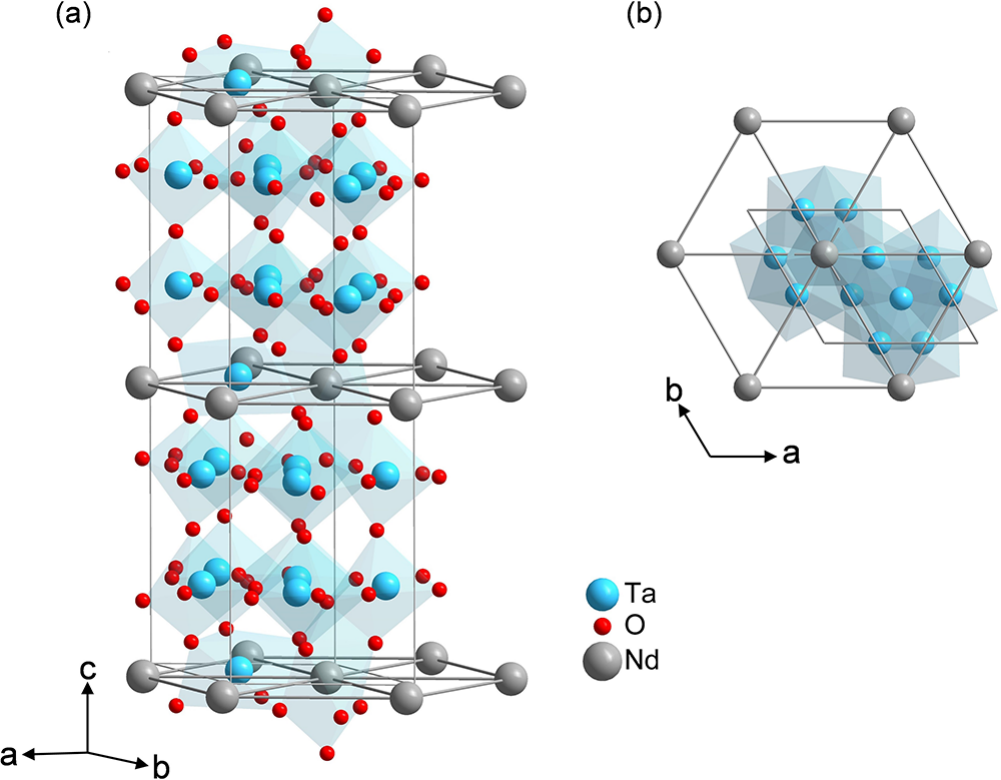
(a) Crystal structure of RETa_7_O_19_ (here RE = Nd). (b) View from the *c*-axis as projected onto the *ab*-plane, showing the perfect triangular lattice within the magnetic layers, separated by two nonmagnetic layers of TaO_7_ polyhedra. The oxygen atoms in (b) are omitted for clarity.

A systematic decrease in the unit cell volume (Supporting Information, Table S2), the magnetic interlayer distances, and the RE–RE distances is observed from RE = Nd to Er, following the decrease in the ionic radii (Supporting Information, Tables S4–S6).^31^ These findings are consistent with the observations on the polycrystalline heptatantalates (see Section 3.1 and Table S1).

The SEM–EDS point analysis was employed for elemental analysis and to identify possible flux inclusions. A representative SEM–EDS point analysis spectrum of a NdTa_7_O_19_ crystal, along with the corresponding SEM image of the crystal (Figure 8a), shows peaks of all the constituent elements without any flux components or other impurities. The average atomic percent values obtained from 11 point analyses are 3.7(1) atom% Nd, 26.0(1) atom% Ta, and 70.4(1) atom% O, perfectly corresponding to the theoretical ratio 1:7:19 and thereby confirming the stoichiometry of the sample. A few dark spots observed on the surface correspond to particles of the main flux component, K_2_Mo_3_O_10_.

**Figure 8:**
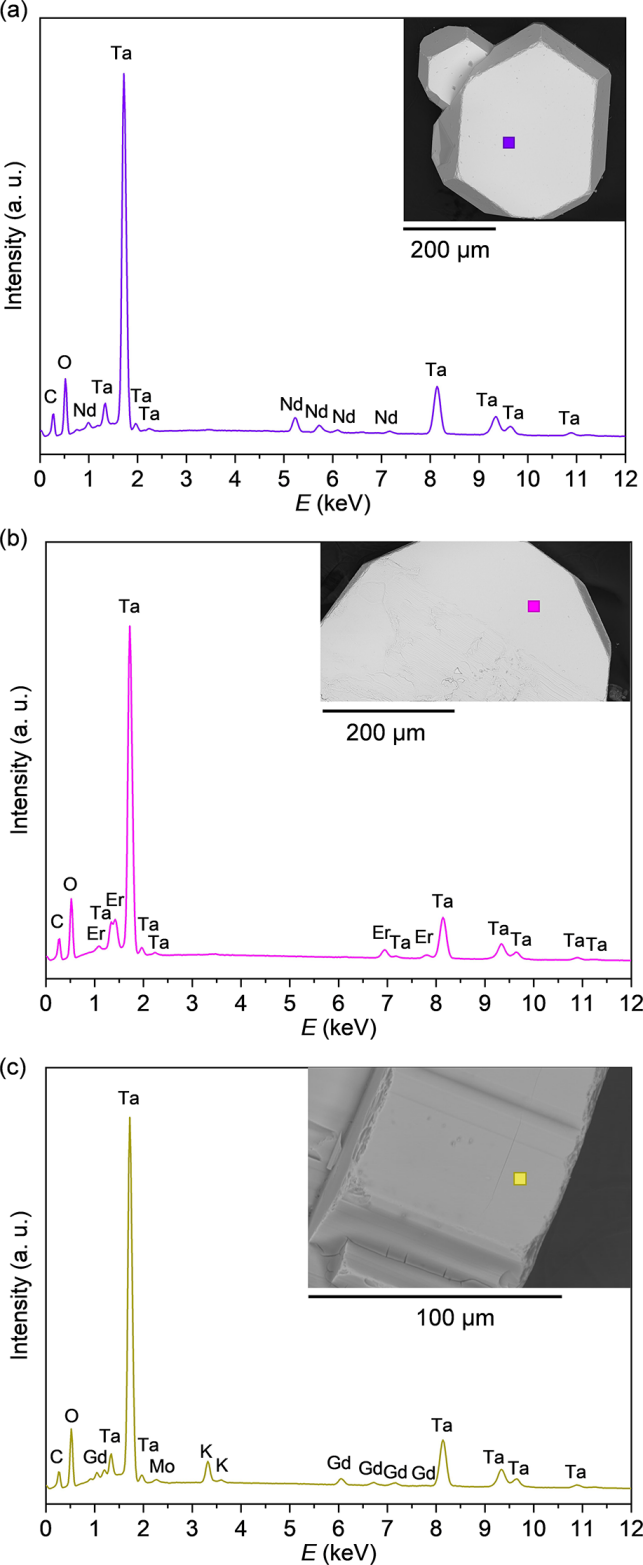
(a) SEM–EDS spectrum of a hexagonal NdTa_7_O_19_ crystal, with the analysis area marked by a violet square, confirming stoichiometric Nd, Ta, and O ratios. (b) SEM–EDS spectrum of an ErTa_7_O_19_ crystal, with the analysis area indicated by a pink square, confirming Er, Ta, and O. (c) SEM–EDS spectrum of a GdTa_7_O_19_ crystal, with the analysis area marked by a yellow square (inset), detecting Gd, Ta, O, and traces of K and Mo (from the residual flux on the surface).

The EDS point analyses of ErTa_7_O_19_ (Figure 8b) and GdTa_7_O_19_ single crystals (Figure 8c) confirm the presence of Er, Gd, Ta, and O. For ErTa_7_O_19_, the average elemental composition from 10 point analyses is 3.5(2) atom % Er, 25.8(1) atom % Ta, and 70.4 atom % of O with trace amounts of K, 0.1(1) atom %, and Mo, 0.2(1) atom %, likely from the flux on the surface. The GdTa_7_O_19_ crystal shows slightly higher flux residues (Figure 8c) with 7-point analyses yielding 3.5(3) atom % Gd, 21.6(7) atom % Ta, 67.1(3) atom % O, 6.3(4) atom % K, and 1.5(6) atom % Mo.

### 3.4 Magnetic Properties

The magnetic susceptibility of NdTa_7_O_19_ (Figure 9a) shows no signs of magnetic ordering down to 2 K, which suggests a possible dynamical magnetic ground state and is consistent with previous results obtained on polycrystalline NdTa_7_O_19_.^5^

**Figure 9:**
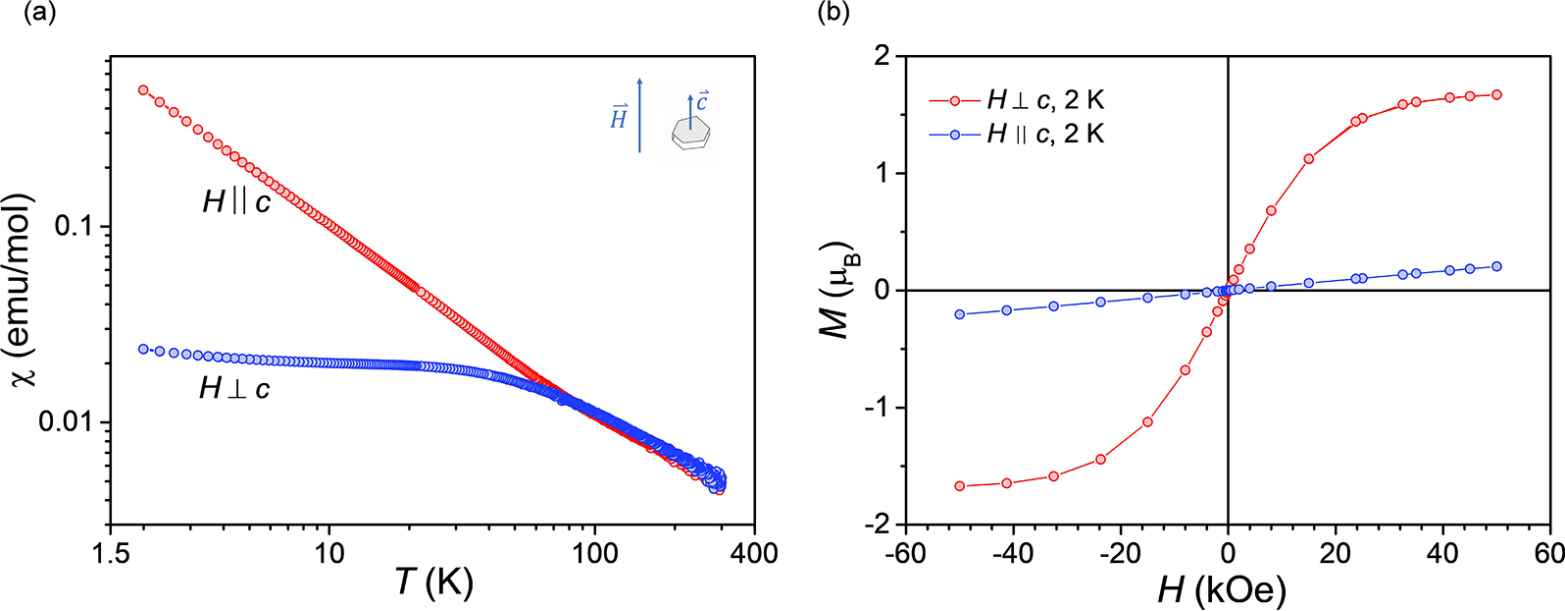
Magnetic susceptibility of a NdTa_7_O_19_ single crystal (a) and *M*(*H*) curves (b), showing a pronounced difference in the magnetic response in both principal crystallographic directions, *H* ∥ *c* and *H* ⊥ *c*, which represents direct evidence of the easy-axis magnetic anisotropy.

When the magnetic field *H* of 1 kOe is applied parallel (*H* ∥ *c*) and perpendicular (*H* ⊥ *c*) to the *c*-axis (Figure 9b), a very similar paramagnetic response is observed for both crystallographic directions as the temperature decreases. However, below 90 K, the magnetic responses of the two orientations become increasingly different in size and behavior. This divergence is evident in both the magnetic susceptibility (Figure 9a) and field-dependent magnetization at 2 K, *M*(*H*) (Figure 9b), providing clear evidence of easy-axis single-ion magnetic anisotropy, as previously suggested by crystal-electric-field modeling of polycrystalline samples.^5^

A similar magnetic response to NdTa_7_O_19_ is also observed in the magnetic susceptibility and field-dependent magnetization of ErTa_7_O_19_ (Figure 10a,b). In the external field of 1 kOe, a paramagnetic behavior is observed in both crystallographic directions at high temperatures to about 150 K. However, as the temperature decreases to 2 K, a significant divergence in the magnetic response for both crystallographic directions is observed, *H* ∥ *c* and *H* ⊥ *c*, again providing evidence of easy-axis-type magnetic anisotropy. The absence of magnetic ordering down to 2 K suggests a similar dynamic magnetic ground state as previously observed for NdTa_7_O_19_.^5^

**Figure 10:**
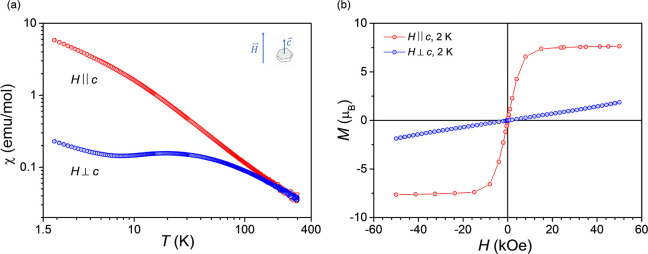
(a) Magnetic susceptibility of a ErTa_7_O_19_ single crystal showing a significant difference in the magnetic response in the crystallographic directions *H* ∥ *c* and *H* ⊥ *c* as a consequence of large magnetic anisotropy. (b) *M*(*H*) curves for *H* ⊥ *c* and *H* ∥ *c* at 2 K. A significant magnetic anisotropy is also observed, similar to (a).

The magnetic response of GdTa_7_O_19_ to a field of 1 kOe (Figure 11a) is substantially different from those of the previous two representatives of the RETa_7_O_19_ series and corresponds to that of an isotropic paramagnet. The results of susceptibility and field-dependent magnetization (Figure 11b) are very similar for both crystallographic directions, showing the absence of anisotropy in the magnetism of this compound. This isotropic magnetic behavior is consistent with findings from a recent study,^35^ which reported rare-earth dependent magnetic properties for six polycrystalline RETa_7_O_19_ compounds RE = Pr, Sm, Eu, Gd, Dy, and Ho.

**Figure 11:**
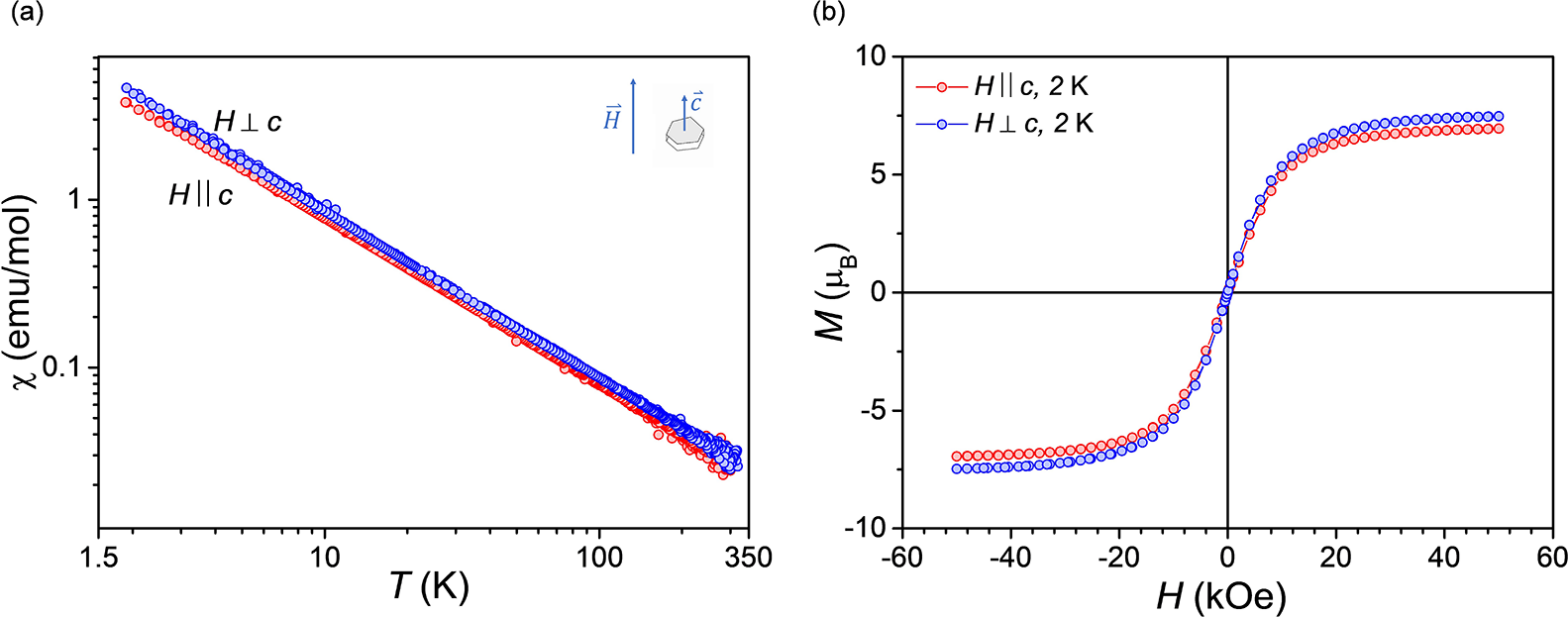
(a) Magnetic susceptibility of a GdTa_7_O_19_ single crystal showing the absence of anisotropy, i.e., a nearly identical paramagnetic response for both *H* ∥ *c*, and *H* ⊥ *c*, typical of a paramagnet. (b) *M*(*H*) curves for *H* ∥ *c* and *H* ⊥ *c* at 2 K, showing a similar magnetic response in both directions, in line with observations in (a).

## 4 Conclusions

High-quality single crystals of NdTa_7_O_19_, the first Ising quantum spin liquid candidate on a triangular lattice, were successfully grown using the K_2_Mo_3_O_10_–B_2_O_3_ flux. With lateral sizes up to 3.5 mm, thicknesses up to 2 mm, and hexagonal, plate-like morphologies, these crystals are the largest reported to date.

To maximize the yield and reduce the formation of secondary neodymium phases, the crystals were grown from polycrystalline NdTa_7_O_19_, obtained by optimizing a previously reported solid-state method.^24^ This optimized flux method was extended to grow large, high-quality single crystals of two additional compounds in the series, ErTa_7_O_19_ and GdTa_7_O_19_.

A substantial easy-axis magnetic anisotropy in NdTa_7_O_19_ was observed in its magnetic susceptibility at low temperatures down to 2 K, providing clear proof of the anisotropy indicated by earlier modeling of the crystal electric field in the polycrystalline sample.^5^ Similarly, ErTa_7_O_19_ also exhibited a pronounced magnetic anisotropy of the same type. No evidence of magnetic ordering is found down to 2 K, suggesting a possible exotic magnetic ground state for this representative. In contrast, GdTa_7_O_19_ showed a paramagnetic behavior, in agreement with findings from polycrystalline samples.^35^

The obtained high-quality single crystals will allow new insight into the exciting magnetism of these frustrated magnets. More detailed and *q*-resolved investigations of the magnetic ground state, magnetic correlations, and excitations are now possible. Furthermore, the flux method holds the potential for successful growth of additional rare-earth heptatantalates in the series.

## Supplementary Material



## Notes

Summary of SCXRD data, data collection and structure refinement details; structural parameters derived from Rietveld refinement fits of polycrystalline NdTa_7_O_19_, ErTa_7_O_19_ and GdTa_7_O_19_ laboratory PXRD data; and additional images of NdTa_7_O_19_ single crystals (PDF)
