# Supplementary material for: Optimized Flux Single-Crystal Growth of the Quantum Spin Liquid Candidate NdTa7O19 and Other Rare-Earth Heptatantalates, ErTa7O19 and GdTa7O19
Source: Cryst Growth Des. 2025 Jun 2;25(12):4646–54. doi: 10.1021/acs.cgd.5c00624 (PMC12186254; doi:10.1021/acs.cgd.5c00624)
Supplement: Supplementary file 1 [file cg5c00624_si_001.pdf]

Supporting Information

Optimized flux single-crystal growth of the quantum spin liquid candidate $NdTa_7O_{19}$ and other rare-earth heptatantalates, $ErTa_7O_{19}$ and $GdTa_7O_{19}$

Lia Šibav[1,2], Matic Lozinšek[1,2], Zvonko Jagličić[3,4], Tina Arh[1,5], Panchanana Khuntia[6,7], Andrej Zorko[1,5] and Mirela Dragomir[1,2*]

[1]*Jožef Stefan Institute, Jamova cesta 39, 1000 Ljubljana, Slovenia*
[2]*Jožef Stefan International Postgraduate School, Jamova cesta 39, 1000 Ljubljana, Slovenia*
[3]*Faculty of Civil and Geodetic Engineering, University of Ljubljana, Jamova cesta 2, 1000 Ljubljana, Slovenia*
[4]*Institute of Mathematics, Physics and Mechanics, Jadranska cesta 19, 1000 Ljubljana, Slovenia*
[5]*Faculty of Mathematics and Physics, University of Ljubljana, 1000 Ljubljana, Slovenia*
[6]*Department of Physics, Indian Institute of Technology Madras, 600036 Chennai, India*
[7]*Quantum Centre of Excellence for Diamond and Emergent Materials, Indian Institute of Technology Madras, 600036 Chennai, India*

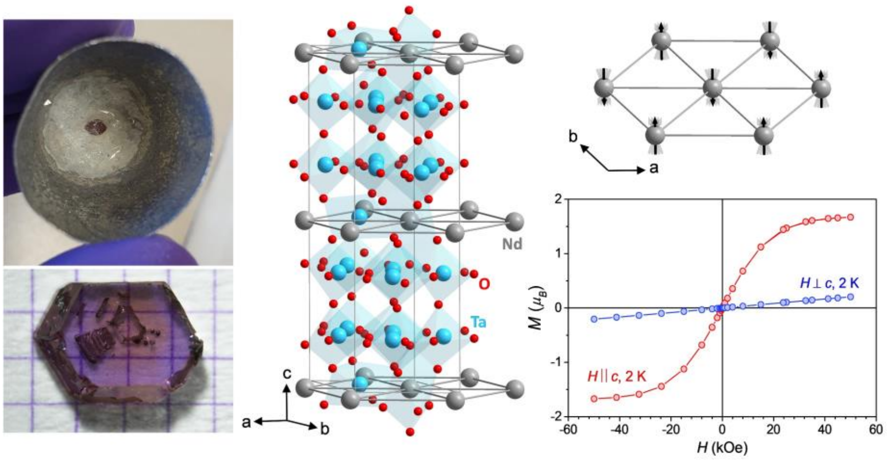

Table S1. A summary of Rietveld refinement analysis results on the polycrystalline $NdTa_7O_{19}$, $ErTa_7O_{19}$ and $GdTa_7O_{19}$. The data was collected at room-temperature using Cu Kα radiation (λ = 1.5406 Å).

	**$NdTa_7O_{19}$**	**$GdTa_7O_{19}$**	**$ErTa_7O_{19}$**
**Space group**	$P\bar{6}c2$	$P\bar{6}c2$	$P\bar{6}c2$
***a*, *b* (Å)**	6.22277(3)	6.21188(4)	6.20178(3)
***c* (Å)**	19.93551(5)	19.89306(8)	19.85693(5)
**V (Å^3)**	668.536(4)	664.780(6)	661.418(5)
**R_{wp} (%)**	3.30	3.45	3.43
**R_{exp} (%)**	2.32	2.35	2.30
**S (R_{wp}/R_{exp})**	1.42	1.47	1.49
**Atom**	*x*/*y*/*z*<br>*U*iso	*x*/*y*/*z*<br>*U*iso	*x*/*y*/*z*<br>*U*iso
R	0.66667/0.33333/0<br>0.0036(6)	0.66667/0.33333/0<br>0.0188(16)	0.66667/0.33333/0<br>0.0057(7)
**Ta1**	0.33333/0.66667/0<br>0.0050	0.33333/0.66667/0<br>0.0023(8)	0.33333/0.66667/0<br>0.0050
**Ta2**	0.63963(25)/0.63957(30)/0.15589(1)<br>0.00318(19)	0.64033(26)/0.63831(29)/0.15576(2)<br>0.00666(26)	0.64019(28)/0.63793(31)/0.15550(1)<br>0.00353(22)
**O1**	0.7391(28)/0.977(5)/0.15315(19)<br>0.0133(15)	0.7372(27)/0.973(5)/0.15324(26)<br>0.0171(21)	0.7305(28)/0.958(5)/0.15198(20)<br>0.0190(18)
**O2**	0.5688(8)/0.6210(10)/0.05810(18)<br>0.0050	0.5690(14)/0.6051(15)/0.05869(23)<br>0.0127(23)	0.5747(12)/0.6121(14)/0.05700(19)<br>0.0104(18)
**O3**	0.6216(14)/0.5838(14)/0.2500<br>0.0050	0.6240(23)/0.5889(25)/0.2500<br>0.015(4)	0.6187(16)/0.5803(15)/0.2500<br>0.0050
**O4**	0.33333/0.66667/0.1639(5)<br>0.0050	0.33333/0.66667/0.1658(5)<br>0.0050	0.33333/0.66667/0.1639(5)<br>0.0050
**O5**	0.66667/0.33333/0.1301(6)<br>0.0068(31)	0.66667/0.33333/0.1263(8)<br>0.022(5)	0.66667/0.33333/0.1271(6)<br>0.006(3)

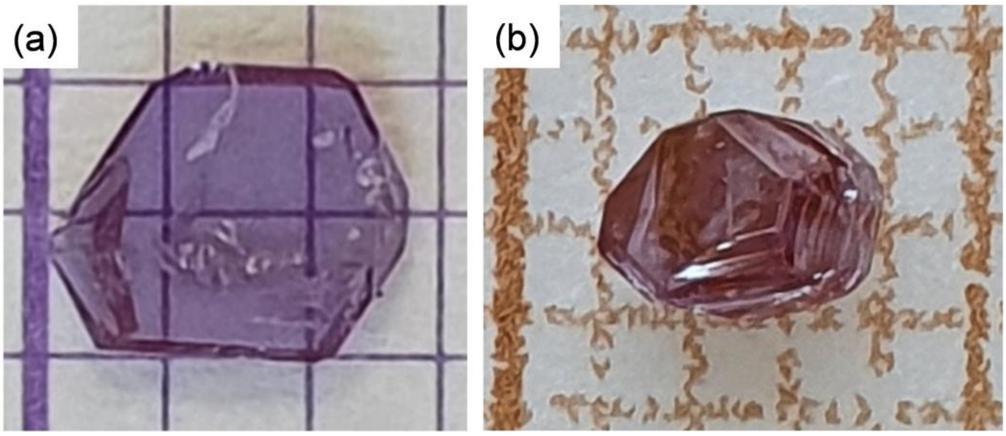

Figure S1. Single crystals of $NdTa_7O_{19}$. (**a**) A plate-like crystal grown with a 9:1 $K_2Mo_3O_{10}/B_2O_3$ flux mass ratio. (**b**) A crystal with larger thickness, grown with a 4:1 $K_2Mo_3O_{10}/B_2O_3$ mass ratio.

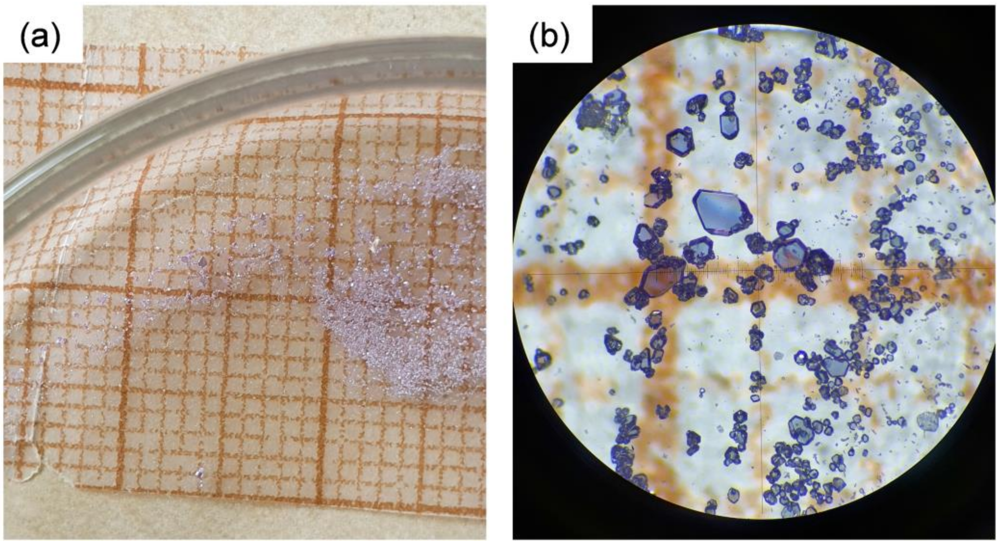

Figure S2. Representative images of small $NdTa_7O_{19}$ single crystals, grown in the process of optimization that did not yield crystals of significant size: (**a**) displayed on a millimeter grid for size reference and (**b**) magnified optical microscope image on a millimeter grid, emphasizing the crystal morphology.

Table S2. Summary of single-crystal X-ray diffraction crystal data, data collection and structure refinement details for the crystal structures of $NdTa_7O_{19}$, $GdTa_7O_{19}$, and $ErTa_7O_{19}$.

**Compound**	**$NdTa_7O_{19}$**	**$GdTa_7O_{19}$**	**$ErTa_7O_{19}$**
M_r	1714.89	1727.90	1737.91
Crystal system	hexagonal	hexagonal	hexagonal
Space group	$P\bar{6}c2$	$P\bar{6}c2$	$P\bar{6}c2$
a [Å]	6.21494(10)	6.20871(10)	6.19718(6)
b [Å]	6.21494(10)	6.20871(10)	6.19718(6)
c [Å]	19.9382(4)	19.9087(4)	19.8613(2)
α [°]	90	90	90
β [°]	90	90	90
γ [°]	120	120	120
V [Å^3]	666.94(3)	664.62(3)	660.581(16)
Z	2	2	2
ρ_{calc} [g/cm^3]	8.539	8.634	8.737
Crystal size [mm^3]	0.090 × 0.077 × 0.050	0.208 × 0.135 × 0.029	0.160 × 0.139 × 0.077
Radiation type	Ag Kα	Ag Kα	Ag Kα
λ [Å]	0.56087	0.56087	0.56087
T [K]	100	100	100
μ [mm^{-1}]	32.772	33.482	34.408
F(000)	1446	1454	1462
θ_{max} [°]	32.324	32.245	30.653
Index ranges	$-11 \leq h \leq 11$<br>$-9 \leq k \leq 11$<br>$-36 \leq l \leq 35$	$-11 \leq h \leq 10$<br>$-11 \leq k \leq 9$<br>$-35 \leq l \leq 37$	$-11 \leq h \leq 10$<br>$-11 \leq k \leq 11$<br>$-36 \leq l \leq 35$
Reflections collected	13542	16495	40463
Independent reflections	1471	1525	1431
Reflections with [$I > 2\sigma(I)$]	1432	1462	1374
R_{int}	0.0473	0.0573	0.1374
R_{sigma}	0.0231	0.0249	0.0335
Data/restraints/parameters	1471/0/45	1525/6/45	1431/6/46
S	1.174	1.158	1.201
R_1, wR_2 [$I > 2\sigma(I)$]	0.0198, 0.0447	0.0196, 0.0408	0.0237, 0.0524
R_1, wR_2 [all data]	0.0208, 0.0450	0.0213, 0.0415	0.0254, 0.0545
$\Delta\rho_{min}$, $\Delta\rho_{max}$ [eÅ^{-3}]	−1.528, 2.546	−2.330, 2.121	−3.816, 3.301
Flack x	−0.010(15)	0.007(14)	BASF = 0.63(4) [a]

[a] refined as an inversion twin

CSD Deposition Numbers 2407250 (for $NaTa_7O_{19}$), 2407251 (for $GdTa_7O_{19}$), and 2407252 (for $ErTa_7O_{19}$) contain the supplementary crystallographic data for this paper. These data can be obtained free of charge from FIZ Karlsruhe via www.ccdc.cam.ac.uk/structures.

The anisotropic thermal ellipsoid of atom O1 in crystal structures of $GdTa_7O_{19}$, and $ErTa_7O_{19}$ was restrained with ISOR 0.001 to prevent it from becoming non-positive definite.

Note: the checkCIF reports show two alerts level A for the $NaTa_7O_{19}$ and four for $ErTa_7O_{19}$. These are caused by large residual electron density minima and maxima. Full single-crystal datasets have been measured on several different crystals of each compound. The aforementioned issues were observed consistently in all analyzed single crystals. Typically, the problems were exacerbated in larger crystals, which indicates possible absorption effects.

Table S3. Fractional atomic coordinates and equivalent isotropic displacement parameters (Å^2) for the crystal structures of $NdTa_7O_{19}$, $GdTa_7O_{19}$, and $ErTa_7O_{19}$.

	Atom	*x*	*y*	*z*	U_{eq}
$NdTa_7O_{19}$	Ta1	0.3333	0.6667	0	0.00588(14)
	Ta2	0.63907(7)	0.63951(6)	0.15602(2)	0.00639(6)
	Nd1	0.6667	0.3333	0	0.0069(2)
	O1	0.7512(10)	1.0016(18)	0.15375(15)	0.0086(5)
	O2	0.5709(8)	0.6222(7)	0.0568(2)	0.0072(6)
	O3	0.6246(10)	0.5791(11)	0.25	0.0068(9)
	O4	0.3333	0.6667	0.1681(4)	0.0064(9)
	O5	0.6667	0.3333	0.1314(4)	0.0072(11)
$GdTa_7O_{19}$	Ta1	0.3333	0.6667	0	0.00573(14)
	Ta2	0.63905(7)	0.63898(6)	0.15581(2)	0.00605(5)
	Gd1	0.6667	0.3333	0	0.0069(2)
	O1	0.7529(10)	1.0037(18)	0.15345(15)	0.0080(5)
	O2	0.5718(7)	0.6194(6)	0.05606(19)	0.0064(6)
	O3	0.6243(9)	0.5791(10)	0.25	0.0081(9)
	O4	0.3333	0.6667	0.1677(4)	0.0073(9)
	O5	0.6667	0.3333	0.1310(4)	0.0091(11)
$ErTa_7O_{19}$	Ta1	0.3333	0.6667	0	0.0091(2)
	Ta2	0.63927(9)	0.63829(8)	0.15558(2)	0.00901(8)
	Er1	0.6667	0.3333	0	0.0097(2)
	O1	0.7524(14)	1.002(3)	0.15316(19)	0.0113(6)
	O2	0.5723(9)	0.6156(8)	0.0553(3)	0.0103(9)
	O3	0.6226(11)	0.5786(13)	0.25	0.0100(12)
	O4	0.3333	0.6667	0.1663(5)	0.0088(12)
	O5	0.6667	0.3333	0.1311(5)	0.0106(14)

Table S4. Selected bond distances (Å) and angles (°) for the crystal structure of $NdTa_7O_{19}$.

Bond distance (Å)			
Nd−O2	2.435(4)	Ta2−O1	1.996(10)
Nd−O5	2.620(8)	Ta2−O2	2.014(4)
Ta1−O2	1.987(4)	Ta2−O3	1.9043(10)
Ta2−O1^{vi}	1.982(10)	Ta2−O4	2.0044(11)
Ta2−O1^{iv}	2.422(4)	Ta2−O5	2.054(2)
Angle (°)			
O2^{v}−Ta1−O2	166.2(2)	O3−Ta2−O5	94.0(3)
O2^{ii}−Ta1−O2	99.9(2)	O4−Ta2−O1^{iv}	64.8(2)
O2^{iii}−Ta1−O2	90.72(17)	O4−Ta2−O2	86.5(2)
O2^{i}−Ta1−O2	80.4(2)	O4−Ta−O5	128.94(5)
O1−Ta2−O1^{iv}	137.8(3)	O5−Ta2−O1^{iv}	64.5(2)
O1^{vi}−Ta2−O1	85.0(2)	O2^{i}−Nd1−O2	63.53(19)
O1^{vi}−Ta2−O1^{iv}	137.1(3)	O2^{ix}−Nd1−O2	100.62(19)
O1−Ta2−O2	89.05(14)	O2^{viii}−Nd1−O2	155.57(19)
O1^{vi}−Ta2−O2	99.58(15)	O2^{vii}−Nd1−O2	100.11(11)
O1−Ta2−O4	73.68(12)	O2−Nd1−O5^{i}	117.72(9)
O1^{vi}−Ta2−O4	157.75(14)	O2−Nd1−O5	62.28(9)
O1^{vi}−Ta2−O5	73.30(13)	Ta2^{x}−O1−Ta2^{iii}	102.8(3)
O1−Ta2−O5	153.57(16)	Ta2−O1−Ta2iii	102.1(3)
O2−Ta2−O1^{iv}	81.41(14)	Ta2^{x}−O1−Ta2	154.9(2)
O2−Ta2−O5	80.1(2)	Ta2^{xi}−O3−Ta2	159.5(3)
O3−Ta2−O1	101.53(19)	Ta2^{iv}−O4−Ta2	118.58(9)
O3−Ta2−O1^{iv}	84.01(18)	Ta2^{vii}−O5−Ta2	114.49(16)
O3−Ta2−O1^{vi}	91.41(18)	Ta2−O2−Nd1	111.94(16)
O3−Ta2−O2	165.4(2)	Ta2−O5−Nd1	103.8(2)
O3−Ta2−O4	86.9(3)	Ta1−O2−Nd1	108.06(17)

Symmetry codes: (i) $-y + 1, -x + 1, -z$; (ii) $x, x - y + 1, -z$; (iii) $-y + 1, x - y + 1, z$; (iv) $-x + y, -x + 1, z$; (v) $-x + y, y, -z$; (vi) $-y + 2, x - y + 1, z$; (vii) $-y + 1, x - y, z$; (viii) $x, x - y, -z$; (ix) $-x + y + 1, y, -z$; (x) $-x + y + 1, -x + 2, z$; (xi) $x, y, -z + 1/2$.

Table S5. Selected bond distances (Å) and angles (°) for the crystal structure of $GdTa_7O_{19}$.

Bond distance (Å)			
Gd1−O2	2.407(3)	Ta2−O1	2.007(9)
Gd1−O5	2.607(8)	Ta2−O2	2.020(4)
Ta1−O2	1.990(3)	Ta2−O3	1.9049(10)
Ta2−O1^{viii}	1.968(9)	Ta2−O4	2.0036(11)
Ta2−O1^{vi}	2.422(3)	Ta2−O5	2.050(2)
Angle (°)			
O2^{iii}−Ta1−O2	99.7(2)	O3−Ta2−O5	94.3(3)
O2^{i}−Ta1−O2	91.60(15)	O4−Ta2−O1^{iv}	65.1(2)
O2^{v}−Ta1−O2	165.3(2)	O4−Ta2−O2	86.8(2)
O2−Ta1−O2^{ii}	78.9(2)	O4−Ta2−O5	129.00(5)
O1^{vi}−Ta2−O1^{iv}	137.0(3)	O5−Ta2−O1^{iv}	64.4(2)
O1^{vi}−Ta2−O1	84.67(18)	O2^{ix}−Gd1−O2	100.22(11)
O1−Ta2−O1^{iv}	138.2(3)	O2^{ii}−Gd1−O2	63.36(17)
O1^{vi}−Ta2−O2	99.24(14)	O2^{viii}−Gd1−O2	155.53(18)
O1−Ta2−O2	89.45(13)	O2^{vii}−Gd1−O2	100.56(18)
O1^{vi}−Ta2−O4	157.58(12)	O2−Gd1−O5^{ii}	117.63(9)
O4−Ta2−O1	73.76(11)	O2−Gd1−O5	62.37(8)
O1^{vi}−Ta2−O5	73.42(11)	Ta2^{xi}−O1−Ta2^{i}	102.9(3)
O1−Ta2−O5	153.33(16)	Ta2^{xi}−O1−Ta2	155.15(17)
O2−Ta2−O1^{iv}	81.29(13)	Ta2−O1−Ta2^{i}	101.7(3)
O2−Ta2−O5	79.5(2)	Ta2−O3−Ta2^{xi}	159.7(3)
O3−Ta2−O1	101.46(18)	Ta2^{i}−O4−Ta2	118.63(9)
O3−Ta2−O1^{iv}	84.05(18)	Ta2^{x}−O5−Ta2	114.37(17)
O3−Ta2−O1^{vi}	91.57(17)	Ta2−O5−Gd1	104.0(2)
O3−Ta2−O2	165.3(2)	Ta2−O2−Gd1	112.37(14)
O3−Ta2−O4	86.9(3)	Ta1−O2−Gd1	108.87(16)

Symmetry codes: (i) $-y+1, x-y+1, z$; (ii) $-y+1, -x+1, -z$; (iii) $x, x-y+1, -z$; (iv) $-x+y, -x+1, z$; (v) $-x+y, y, -z$; (vi) $-y+2, x-y+1, z$; (vii) $-x+y+1, y, -z$; (viii) $x, x-y, -z$; (ix) $-x+y+1, -x+1, z$; (x) $-y+1, x-y, z$; (x) $-x+y+1, -x+2, z$; (xi) $x, y, -z+1/2$.

Table S6. Selected bond distances (Å) and angles (°) for the crystal structure of $ErTa_7O_{19}$.

Bond distance (Å)			
Er1−O2	2.373(4)	Ta2−O1	1.997(13)
Er1−O5	2.603(10)	Ta2−O2	2.024(5)
Ta1−O2	1.993(5)	Ta2−O3	1.9042(12)
Ta2−O1^{viii}	1.972(13)	Ta2−O4	2.0011(13)
Ta2−O1^{iv}	2.419(4)	Ta2−O5	2.039(3)
Angle (°)			
O2−Ta1−O2^{ii}	92.5(2)	O3−Ta2−O5	94.4(3)
O2−Ta1−O2^{i}	99.9(3)	O4−Ta2−O1^{ii}	64.8(3)
O2−Ta1−O2^{iii}	77.3(3)	O4−Ta2−O2	86.6(3)
O2−Ta1−O2^{v}	164.2(3)	O4−Ta2−O5	128.94(7)
O1−Ta2−O1^{ii}	137.7(4)	O5−Ta2−O1^{ii}	64.7(3)
O1^{vi}−Ta2−O1	84.4(2)	O2^{viii}−Er1−O2	100.7(2)
O1^{vi}−Ta2−O1^{ii}	137.7(4)	O2^{vii}−Er1−O2	155.4(2)
O1^{vi}−Ta2−O2	99.00(18)	O2−Er1−O2^{iii}	63.2(2)
O1−Ta2−O2	89.93(16)	O2^{ix}−Er1−O2	100.27(15)
O1^{vi}−Ta2−O4	157.35(16)	O2^{iii}−Er1−O5	117.59(12)
O1−Ta2−O4	73.58(15)	O2−Er1−O5	62.41(12)
O1−Ta2−O5	153.5(2)	Ta2^{x}−O1−Ta2	155.4(2)
O1^{vi}−Ta2−O5	73.71(15)	Ta2^{x}−O1−Ta2^{iv}	102.2(4)
O2−Ta2−O1^{ii}	81.00(17)	Ta2−O1−Ta2^{iv}	102.2(4)
O2−Ta2−O5	79.1(3)	Ta2−O3−Ta2^{xi}	160.0(4)
O3−Ta2−O1^{ii}	84.0(2)	Ta2^{ii}−O4−Ta2	118.88(10)
O3−Ta2−O1	101.4(2)	Ta2^{ix}−O5−Ta2	114.5(2)
O3−Ta2−O1^{vi}	92.0(2)	Ta2−O2−Er1	112.90(19)
O3−Ta2−O2	165.0(3)	Ta2−O5−Er1	103.8(3)
O3−Ta2−O4	87.2(3)	Ta1−O2−Er1	109.8(2)

Symmetry codes: (i) $x, x-y+1, -z$; (ii) $-x+y, -x+1, z$; (iii) $-y+1, -x+1, -z$; (iv) $-y+1, x-y+1, z$; (v) $-x+y, y, -z$; (vi) $-y+2, x-y+1, z$; (vii) $x, x-y, -z$; (viii) $-x+y+1, y, -z$; (ix) $-y+1, x-y, z$; (x) $-x+y+1, -x+2, z$; (xi) $x, y, -z+1/2$.
